# Diabetic retinopathy: a neuropathy

**DOI:** 10.31744/einstein_journal/2021ED6110

**Published:** 2020-12-17

**Authors:** Thiago Gonçalves dos Santos Martins

**Affiliations:** 1 Universidade de Coimbra Coimbra Portugal Universidade de Coimbra, Coimbra, Portugal.

Diabetic retinopathy is the main cause of blindness in the working-age population, in the Western world. Classically, it is described as a microcirculatory disease, but evidence shows that there are neurodegenerative manifestations that precede the vascular manifestations, such as nervous changes, even in the absence of a pericyte lesion, previously considered the first sign of diabetic retinopathy.^(1)^ Major neurodegenerative changes include apoptosis and glial activation, found in retinas of diabetic donors with no documented vascular changes prior to ophthalmic examinations.^(2)^ Thus, the documentation of patients merely with retinography and fundoscopy may not detect early changes in diabetic retinopathy.

## Neurodegeneration

The pathophysiology of diabetic retinopathy is related to the apoptosis of ganglion and amacrine and Müller cells of the retina, due to an accumulation of glutamate, which leads to a picture of neurodegeneration and reduction of the layer of nerve fibers and ganglion cells.^(3)^

The pathophysiology of glutamate accumulation is related to dysfunction of the glutamine-synthetase enzyme of Müller cells, which reduces its ability to oxidize glutamate and to remove retinal glutamate by the glia cells. An increase in glutamate concentration leads to cell death due to an intracellular increase in calcium. Diabetes also induces activation of microglia cells, located inside the retina, which migrate to the subretinal space and release cytokines, contributing to neuronal cell death.^(4)^

Hyperglycemia triggers glycosylation of proteins and lipids, which leads to a condition of neurodegeneration, along with ischemic changes that decrease blood supply to the nerves.

It has also been found that the flow is associated with areas of neuropathy. Neurodegenerative abnormalities in areas without vascularization, such as the cornea, proven with confocal microscopy tests in diabetic patients, characterize the independent mechanism of neurodegenerative modification of the vascular change.^(5)^

However, microcirculatory changes may be related to neurological changes, as endothelial changes in the vascular basal membrane may lead to apoptosis of the pericyte in addition to capillary occlusion of the optic nerve capillaries.^(6)^

Research on diabetic neurodegeneration may explain why proliferative diabetic retinopathy and other complications can develop in 20% of diabetic patients kept under strict metabolic control, demonstrating that there are likely other risk factors that need to be controlled.^(7)^It is important, however, to emphasize that there is a greater loss of the nerve fiber layer in highly myopic and elderly patients.^(8)^

## Perspectives

Neuroprotective treatment research can be useful in the management of diabetic retinopathy. Topical use of brimonidine tartrate and somatostatin eye drops cause local vasodilation in the retina, and by increasing blood flow in the retina, can prevent the progression of diabetic retinopathy. Somatostatin is usually reduced in diabetic patients with ganglion cell damage, and may have an impact on neurodegeneration prevention, by reducing glutamate cell accumulation. It also acts by preventing neovascularization and inhibiting vascular endothelial growth factor (VEGF) production.^(9)^An ascorbic acid *deficit* in the vitreous of diabetic patients has been demonstrated. It is an antioxidant substance inhibiting the production of VEGF, and it is important to maintain high levels of this substance in the vitreous of diabetic patients.^(10)^ The pigment epithelium-derived factor (PEDF) is another potent neuroprotective and antiangiogenic element that protects neurons from glutamate-mediated neurodegeneration and is diminished in diabetic retinopathy.^(11)^ Other neuroprotective factors, such as insulin, neuroprotectin D1, brain-derived neurotrophic factor, glial cell line-derived neurotrophic factor, ciliary neurotrophic factor, nerve growth factor, and adrenomedullin may also be involved in the neurodegenerative process that occurs in diabetic retinopathy, but other specific studies still need to be performed.

This could prevent more invasive treatment in the final stages of diabetic retinopathy, using intravitreous antiangiogenic injections and laser photocoagulation in the retina. In addition, the study of the retinal ganglion cell layer in diabetic patients could be a method of early control of disease progression. The confirmation of a neurodegenerative disease can instigate new perspectives of diabetes diagnosis and treatment.

## CONCLUSION

Neurodegeneration can be demonstrated in studies documenting the thickness of nerve fiber and ganglion cell layers as an event preceding vascular changes in diabetic retinopathy. Advances in these studies and in the treatment with neuroprotective drugs can improve the diagnosis and treatment of diabetic retinopathy.
