# Supplementary material for: Diabetic retinopathy: a neuropathy
Source: Einstein (Sao Paulo). 2020 Dec 17;19:eED6110. doi: 10.31744/einstein_journal/2021ED6110 (PMC7793122; doi:10.31744/einstein_journal/2021ED6110)
Supplement: Translation

# Translation: Retinopatia diabética: uma neuropatia

**Authors:** Thiago Gonçalves dos Santos Martins

**Affiliations:** 1 Portugal Universidade de Coimbra, Coimbra, Portugal.

A retinopatia diabética é a principal causa de cegueira na faixa etária de trabalho no mundo ocidental. Classicamente é descrita como uma doença microcirculatória, mas evidências demonstram que há manifestações de neurodegeneração que precedem as manifestações vasculares, ocorrendo alterações nervosas, mesmo na ausência de lesão de pericitos, antes considerado o primeiro sinal da retinopatia diabética.^(1)^ As principais alterações da neurodegeneração incluem apoptose e ativação glial, encontradas em retinas de doadores diabéticos sem alterações vasculares documentadas antes dos exames oftalmológicos.^(2)^ Assim, a documentação de pacientes apenas com exames de retinografia e fundoscopia pode não detectar alterações precoces da retinopatia diabética.

## Neurodegeneração

A fisiopatologia da retinopatia diabética está relacionada com a apotose de células ganglionares, amácrinas e células de Müller da retina, devido a um acúmulo de glutamato, que leva a um quadro de neurodegeneração e redução da camada de fibras nervosas e células ganglionares.^(3)^

A fisiopatologia do acúmulo de glutamato está relacionada com a disfunção da enzima glutamina-sintetase das células de Müller, que reduz sua capacidade de oxidar o glutamato e a retirada reduzida de glutamato retiniano pelas células gliais. O aumento da concentração de glutamato leva à morte celular, devido a um aumento intracelular de cálcio. O diabetes também induz a ativação de células microgliais, localizadas na parte interna da retina, que migram para o espaço sub-retiniano e liberam citocinas que contribuem para a morte celular neuronal.^(4)^

A hiperglicemia desencadeia a glicosilação de proteínas e lipídios, que levam a um quadro de neurodegeneração, junto com alterações isquêmicas que diminuem o suprimento sanguíneo dos nervos.

Também se descobriu que o fluxo está associado a áreas de neuropatia. Alterações neurodegenerativas em locais sem vascularização, como a córnea, comprovadas com exames de microscopia confocal em pacientes diabéticos, caracterizam o mecanismo independente da alteração neurodegenerativa da alteração vascular.^(5)^

Contudo, as alterações microcirculatórias podem estar relacionadas com as alterações neurológicas, já que alterações endoteliais na membrana basal vascular podem levar a apotose de pericito, além da oclusão capilar dos capilares do nervo óptico.^(6)^

Pesquisas sobre neurodegeneração do diabetes podem explicar por que a retinopatia diabética proliferativa e outras complicações podem se desenvolver em 20% dos pacientes diabéticos mantidos com rigoroso controle metabólico, demonstrando que é provável que existam outros fatores de risco que precisam ser controlados.^(7)^ É importante, contudo, reforçar que existe uma perda de camada de fibras nervosas maior em pacientes alto míopes e idosos.^(8)^

## Perspectivas

A pesquisa de tratamento neuroprotetor pode ser útil na conduta da retinopatia diabética. O uso tópico de colírios de tartarato de brimonidina e somatostatina causou uma vasodilatação local na retina, que, por aumentar o fluxo sanguíneo na retina, pode evitar a progressão da retinopatia diabética. A somatostatina costuma estar reduzida em pacientes diabéticos com lesão das células ganglionares, podendo ter uma ação na prevenção de neurodegeneração, reduzindo o acúmulo celular de glutamato. Também atua prevenindo a neovascularização e inibindo a produção de fator de crescimento endotelial vascular (VEGF).^(9)^ Foi demonstrado que um *deficit* de ácido ascórbico no vítreo de pacientes diabéticos. Ele é uma substância antioxidante inibindo a produção de VGEF, sendo dessa forma importante manter alto os níveis dessa substância no vítreo de pacientes diabéticos.^(10)^ O fator derivado do epitélio pigmentar (PEDF) é outro potente fator neuroprotetor e antiangiogênico que está diminuído na retinopatia diabética e protege os neurônios da neurodegeneração mediada pelo glutamato.^(11)^ Outros fatores neuroprotetores, como insulina, neuroprotectina D1, fator neurotrófico derivado do cérebro, fator neurotrófico derivado da linha celular glial, fator neurotrófico ciliar, fator de crescimento nervoso, e adrenomedulina, também podem estar envolvidos no processo neurodegenerativo que ocorre na retinopatia diabética, mas outros estudos específicos precisam ser realizados.

Isso poderia impedir um tratamento mais invasivo nos estágios finais da retinopatia diabética, usando injeções antiangiogênicas intravítreas e fotocoagulação a laser na retina. Também o estudo da camada de células ganglionares da retina de pacientes diabéticos poderia ser método de controle precoce da progressão da doença. A confirmação de uma doença neurodegenerativa pode começar novas perspectivas de diagnóstico e tratamento do diabetes.

## CONCLUSÃO

A neurodegeneração pode ser demonstrada em estudos com documentação da espessura da camada de fibras nervosas e células ganglionares como um evento que precede alterações vasculares na retinopatia diabética. Avanços nesses estudos e no tratamento de drogas neuroprotetoras podem melhorar o diagnóstico e o tratamento da retinopatia diabética.
